# Comparative Effectiveness of Continuous Versus Intermittent Negative Pressure Wound Therapy in the Management of Diabetic Foot Ulcers: A Prospective Observational Study

**DOI:** 10.7759/cureus.89340

**Published:** 2025-08-04

**Authors:** Ajay Nayagam P, Karthikeyan Selvaraj, Srinivasan C

**Affiliations:** 1 General Surgery, Sree Balaji Medical College and Hospital, Chennai, IND

**Keywords:** bates-jensen score, continuous npwt, diabetic foot ulcer, intermittent npwt, negative pressure wound therapy, wound healing

## Abstract

Introduction

Diabetic foot ulcers (DFUs) are a serious complication of diabetes mellitus, contributing to increased morbidity, risk of amputation, and healthcare burden. Negative pressure wound therapy (NPWT) has emerged as a cornerstone in DFU management, but the comparative effectiveness of continuous versus intermittent NPWT remains unclear. This prospective observational study compares continuous and intermittent NPWT in patients with Wagner Grade 1-2 DFUs, assessing healing time (primary outcome), infection recurrence, and amputation rates (secondary outcomes).

Methods

This prospective observational study enrolled 110 patients with DFUs in the Department of General Surgery at Sree Balaji Medical College and Hospital, a tertiary care center in Chennai, India. Patients were divided into two groups, i.e., 55 received continuous NPWT and 55 received intermittent NPWT, based on inpatient registration numbers. Wound healing was assessed using the Bates-Jensen Wound Assessment Tool (BWAT) at baseline and weekly intervals. Key variables analyzed included healing time, ulcer progression, infection recurrence, and amputation rates. Statistical analysis involved t-tests, chi-square tests, logistic regression, and Kaplan-Meier survival analysis.

Results

The mean healing time was significantly lower in the continuous NPWT group (35.2 ± 9.4 days) compared to the intermittent group (42.7 ± 10.5 days, p < 0.001). Logistic regression identified infection absence (OR: 3.21, p < 0.001), Wagner grade ≤2 (OR: 2.87, p < 0.001), HbA1c <8% (OR: 2.15, p = 0.002), and regular use of protective footwear (OR: 1.87, p = 0.013) as independent predictors of successful healing. Continuous NPWT was associated with fewer wound failures and lower amputation rates. *Staphylococcus aureus *was the most commonly isolated organism from infected wounds.

Conclusion

Continuous NPWT demonstrates superior outcomes in wound healing, infection control, and amputation prevention compared to intermittent NPWT in DFU patients. Optimizing patient-specific factors such as glycemic control, infection status, and ulcer grading further enhance therapeutic efficacy.

## Introduction

Diabetic foot ulcers (DFUs) are among the most challenging chronic complications of diabetes mellitus, affecting an estimated 6.3% of people with diabetes globally [1]. In India, prevalence rates are as high as 10.4% among diabetic patients, exacerbated by delayed care, poor glycemic control, and limited access to multidisciplinary wound management teams [1]. DFUs increase the risk of lower extremity amputation by 10 to 20 times and represent a major cause of hospitalization [2]. The pathophysiology involves neuropathy, peripheral vascular disease, infection, and impaired wound healing [3].

Despite negative pressure wound therapy (NPWT)’s established role, optimal delivery, whether continuous or intermittent, remains debated [4]. This prospective observational study aims to compare healing times between continuous and intermittent NPWT, identify predictors of healing (e.g., glycemic control and infection status), and assess secondary outcomes including amputation rates and infection recurrence [5].

Although NPWT’s benefits are well established, there remains debate over optimal delivery modalities. Continuous NPWT delivers constant suction, while intermittent NPWT alternates negative pressure cycles. Intermittent therapy may better stimulate cellular activity by mimicking physiological conditions; however, evidence supporting its superiority is inconclusive. A few randomized studies have compared modes, but results vary depending on ulcer severity, infection status, and patient comorbidities.

## Materials and methods

This prospective observational study was conducted in the Department of General Surgery at Sree Balaji Medical College and Hospital, Chennai, over a period of 18 months from March 2023 to September 2024. The primary objective was to compare the mean healing time between continuous and intermittent NPWT. Secondary objectives included evaluating infection recurrence, amputation rates, and identifying independent predictors of healing, such as glycemic control and ulcer grade. A total of 110 patients were enrolled after obtaining informed written consent. Patients aged between 20 and 75 years, with Wagner Grade 1 or 2 DFUs and ulcer size ranging between 5 cm² and 50 cm², were included. Exclusion criteria comprised venous insufficiency ulcers, malignant wounds, patients on chemotherapy, immunosuppressive agents, or long-term corticosteroids, and those with severe cardiopulmonary or immunocompromised conditions. Baseline investigations such as complete blood count (CBC), renal function tests (RFTs), fasting and postprandial blood sugar (FBS and PPBS), HbA1c, X-ray of the foot, and wound swab culture were performed for all patients.

This study was approved by the Institutional Human Ethics Committee of Sree Balaji Medical College and Hospital, Chennai (approval no.: 002/SBMCH/IHEC/2023/1917). Written informed consent was obtained from all participants prior to inclusion in the study, in accordance with the ethical standards of the 1964 Declaration of Helsinki and its later amendments.

Patients were divided into two groups of 55 each: the continuous NPWT group and the intermittent NPWT group. Group allocation was based on the last digit of the inpatient registration number: patients with even numbers were assigned to the continuous group and those with odd numbers to the intermittent group. All patients received NPWT using the VAC® Therapy system (3M+KCI, USA) with polyurethane foam dressings. The negative pressure was set to 100-125 mmHg, applied continuously or intermittently based on group allocation. Intermittent NPWT was administered in cycles of five minutes of suction followed by two minutes off, repeated throughout the 24-hour period. In the continuous group, constant negative pressure was maintained throughout the therapy, while in the intermittent group, suction was applied cyclically. Dressings were changed every four to five days, and wounds were assessed every three days using the Bates-Jensen Wound Assessment Tool (BWAT). All patients were provided with removable offloading devices tailored to the ulcer location. Infections were managed with culture-guided antibiotics, and surgical debridement was performed every 48-72 hours or as clinically indicated. The primary outcome was the time to complete wound healing, while secondary outcomes included improvement in BWAT score, need for amputation, recurrence of infection, rehospitalization, and number of surgical debridements required. Outcome assessors, including those scoring wound healing via the BWAT, were blinded to the NPWT modality assigned to minimize bias. Complete healing was defined as 100% epithelialization of the wound surface without drainage for two consecutive assessments.

The sample size was calculated using the formula n=2×(Z1−α/2+Z1−βδ/σ)2n=2×(δ/σZ1−α/2​+Z1−β​​)2, where Z1−α/2=1.96Z1−α/2​=1.96 (for 95% confidence), Z1−β=0.84Z1−β​=0.84 (for 80% power), δ (the expected difference in mean healing time between groups) = 7 days, and σ (standard deviation) = 10 days. The expected difference in healing time (δ = 7 days) was based on prior comparative studies [3,4]. Accounting for a potential dropout rate of 10%, the required sample size was increased from 48 to 55 per group to maintain statistical power. Data were entered in Microsoft Excel and analyzed using SPSS version 25. Continuous variables were presented as mean ± standard deviation and compared using independent t-tests. Categorical variables were compared using the Chi-square or Fisher’s exact test. Binary logistic regression was applied to identify independent predictors of successful wound healing, with a p-value <0.05 considered statistically significant.

## Results

A total of 110 patients were enrolled in the study and equally allocated to the continuous and intermittent NPWT groups (n = 55 each). Table 1 shows the baseline demographic and clinical characteristics of both groups. The mean age was comparable (60.8 ± 12.5 vs. 61.2 ± 12.3 years), as were the durations of diabetes, HbA1c levels, and ulcer grades. However, the continuous NPWT group demonstrated a significantly faster mean healing time (35.2 ± 9.4 days) compared to the intermittent group (42.7 ≤ 10.5 days), with a p-value <0.001.

**Table 1:** Demographic and clinical characteristics NPWT: negative pressure wound therapy

Variable	Continuous NPWT (n = 55)	Intermittent NPWT (n = 55)
Age (years)	60.8 ± 12.5	61.2 ± 12.3
Duration of diabetes (yrs)	9.8 ± 4.7	9.6 ± 4.5
HbA1c (%)	8.1 ± 1.7	8.3 ± 1.6
Wagner grade	2.0 ± 0.9	2.1 ± 0.8
Ulcer size (cm²)	5.6 ± 1.8	5.8 ± 1.9
Time to healing (days)	35.2 ± 9.4	42.7 ± 10.5

Independent t-test analysis (Table 2) revealed that time to healing was significantly shorter in the continuous NPWT group (t = -4.51, p < 0.001), while other variables showed no significant difference.

**Table 2:** Independent t-test comparison

Variable	t-statistic	p-value
Time to Healing	-4.51	<0.001
HbA1c (%)	-0.94	0.349
Ulcer Size	-0.57	0.567

A chi-square analysis (Table 3) indicated that infection status (p = 0.013), smoking status (p = 0.020), and alcohol use (borderline, p = 0.052) were significantly associated with NPWT outcomes.

**Table 3:** Chi-square test results

Variable	χ² value	p-value	Interpretation
Infection status	6.12	0.013	Significant
Smoking status	5.45	0.020	Significant
Alcohol use	3.78	0.052	Borderline significant

Logistic regression (Table 4) showed that absence of infection (OR = 3.21, p < 0.001), Wagner grade ≤2 (OR = 2.87, p < 0.001), and non-smoking status (OR = 1.98, p = 0.018) were strong independent predictors of successful wound healing.

**Table 4:** Logistic regression analysis

Predictor	Odds ratio (OR)	95% CI	p-value
Infection absent	3.21	1.65–6.25	<0.001
Wagner Grade ≤2	2.87	1.52–5.43	<0.001
Non-smoker	1.98	1.12–3.49	0.018

A final regression model (Table 5) strengthened the impact of regular footwear use (OR = 1.87, p = 0.013) and HbA1c <8% (OR = 2.15, p = 0.002) on NPWT success. *Staphylococcus aureus *was the most commonly isolated organism from infected wounds.

**Table 5:** Predictors of NPWT success NPWT: negative pressure wound therapy

Predictor	Odds Ratio	95% CI	p-value
Ulcer grade ≤2	3.42	1.89–6.18	<0.001
HbA1c <8%	2.15	1.32–3.51	0.002
Regular footwear use	1.87	1.14–3.07	0.013

## Discussion

Our study demonstrates that continuous NPWT led to faster and more reliable wound healing than intermittent NPWT in patients with DFUs. This finding aligns with previous high-quality studies and multicenter trials supporting the benefits of continuous suction modes.

**Figure 1:**
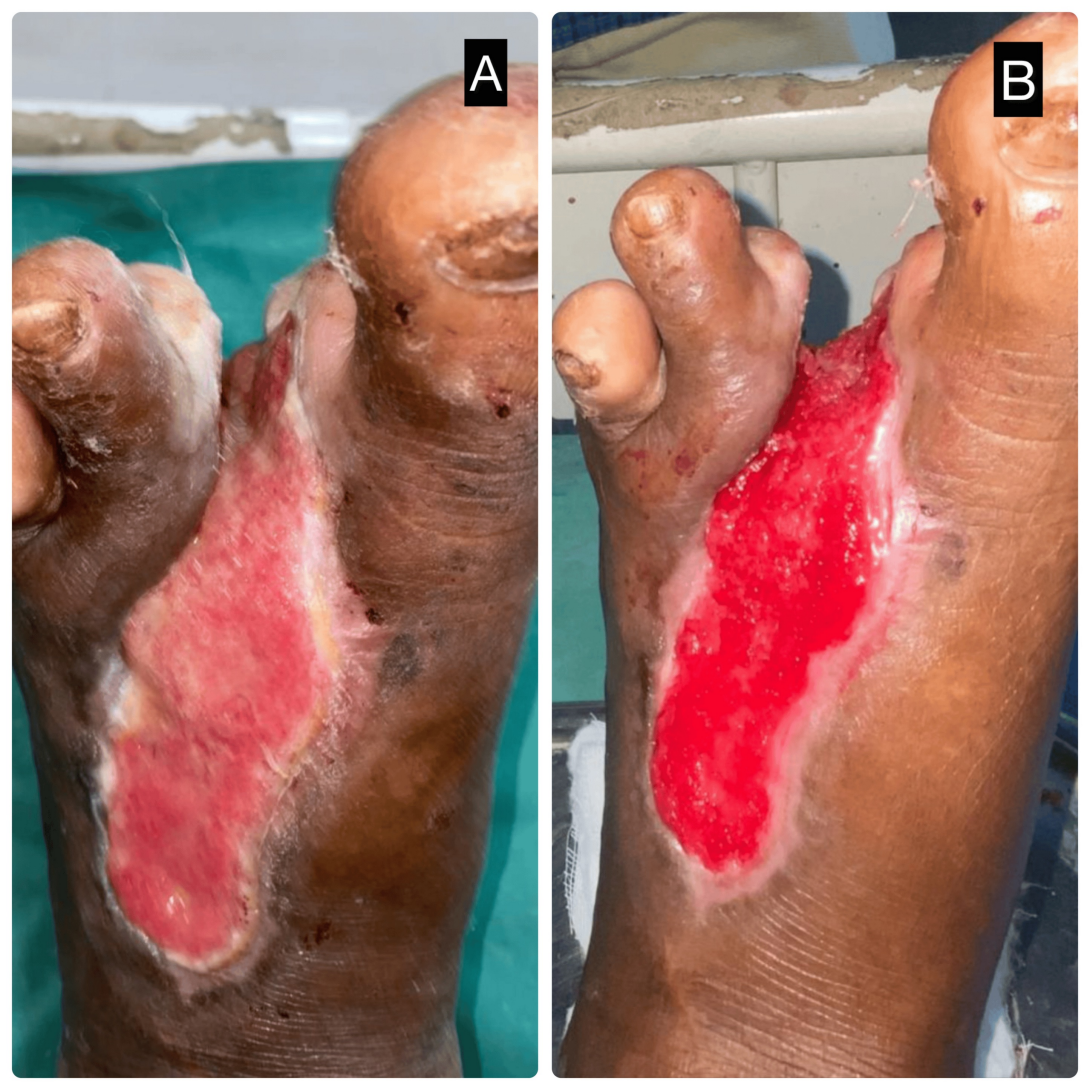
Clinical images showing pre- and post-treatment wound bed status in a representative case of a 52-year-old male with a diabetic foot ulcer treated with continuous negative pressure wound therapy (NPWT). (A) Pre-treatment image showing a slough-covered, shallow ulcer over the dorsum of the forefoot with irregular margins and minimal granulation tissue. (B) Post-treatment image following continuous NPWT for seven days, demonstrating a healthy, well-vascularized granulation tissue bed with clean margins, indicating excellent wound bed preparation and progression toward healing.

**Figure 2:**
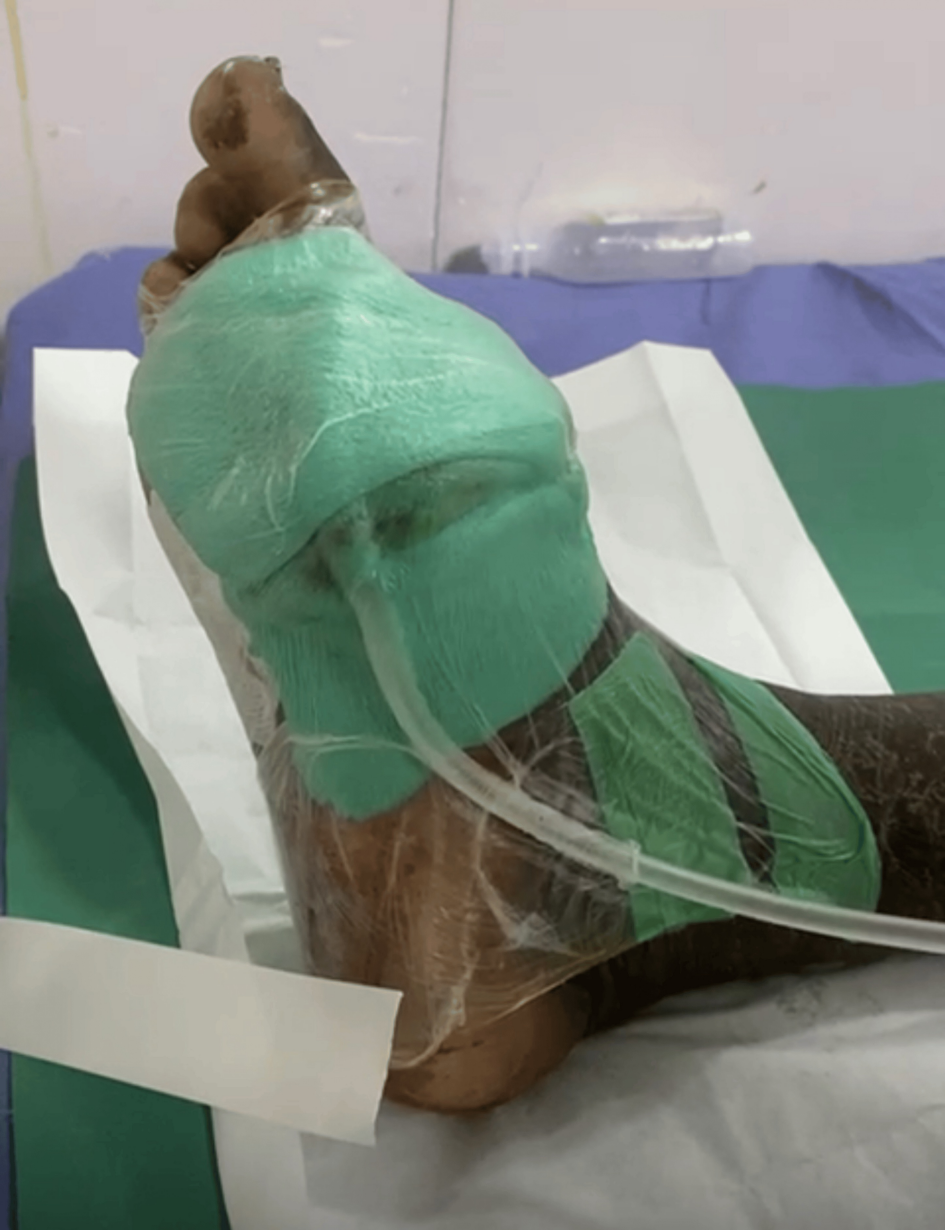
Application of continuous NPWT in the same representative 52-year-old male with a diabetic foot ulcer. NPWT: negative pressure wound therapy

A 52-year-old male with poorly controlled type 2 diabetes mellitus presented with a non-healing ulcer over the dorsum of the left forefoot, persisting for three weeks. On examination, the ulcer was shallow with slough and surrounding induration, without signs of deep infection or osteomyelitis. After initial surgical debridement and optimization of glycemic status, the patient was initiated on continuous NPWT. The dressing was applied using sterile foam, sealed with an occlusive drape, and connected to a continuous suction device maintaining a negative pressure of -125 mmHg. The dressing was left in place for seven days without interruption. Figure 1A shows the slough-covered ulcer prior to therapy, while Figure 1B shows the wound bed post-therapy, with healthy granulation tissue and clean margins. Figure 2 depicts the NPWT dressing in situ, illustrating the closed negative pressure system used during treatment.

A pivotal randomized controlled trial by Armstrong et al. showed significantly reduced healing time and complication rates in patients receiving NPWT after partial foot amputation, establishing it as a superior wound management option over moist wound therapy [1]. This was further supported by Blume et al., who conducted a multicenter randomized trial comparing NPWT with moist wound therapy and found a significantly higher wound closure rate in the NPWT group [2]. Apelqvist et al. analyzed the cost-effectiveness of NPWT and reported that despite higher initial costs, overall expenses were reduced due to faster healing and fewer complications [3]. In addition, the use of Dermagraft, a cultured human dermis, has been shown by Gentzkow et al. to facilitate healing in DFUs, highlighting the role of advanced wound care products alongside NPWT [4].

Similarly, Veves et al. demonstrated that Graftskin, a human skin equivalent, significantly improved healing in noninfected neuropathic DFUs, suggesting a synergy between skin substitutes and NPWT in carefully selected cases [5]. Growth factor therapy also plays a complementary role; Steed showed that recombinant human PDGF enhances healing in lower extremity ulcers, especially when used in a clean wound environment provided by NPWT [6].

Marston et al. reinforced these findings by confirming the safety and efficacy of Dermagraft in a prospective trial, further establishing the benefit of combining biological therapies with NPWT [7]. The importance of pressure offloading was emphasized by Lavery et al., who showed that reducing dynamic foot pressure in high-risk diabetic patients significantly decreased ulcer progression [8]. This was supported by Fleischli et al., who compared various pressure-reducing strategies and concluded that effective offloading is critical to healing outcomes, especially when combined with NPWT [9]. In a randomized evaluation, Eginton et al. showed that NPWT dressings led to faster wound healing and fewer infections compared to standard gauze dressings, confirming its clinical superiority [10].

The mechanisms of NPWT were detailed by Banwell, who explained that topical negative pressure promotes wound contraction, fluid clearance, and granulation tissue formation, providing a scientific basis for its use [11]. Adjunct therapies like electrical stimulation have also been explored; Peters et al. demonstrated its effectiveness in accelerating DFU healing, especially when combined with other modalities like NPWT [12].

Wound severity assessment remains critical; Wagner introduced a grading system that continues to guide DFU management and has shown that Wagner grade ≤2 ulcers respond well to early intervention, including NPWT [13]. On a cellular level, Saxena et al. illustrated how NPWT induces microdeformation of wounds, promoting keratinocyte proliferation and angiogenesis-key steps in tissue repair [14]. Lastly, Greene et al. reported that microdeformational wound therapy significantly increases angiogenesis and modulates matrix metalloproteinases, explaining the enhanced healing seen in NPWT-treated chronic wounds [15].

This study has several limitations. First, it is observational in design, limiting the ability to establish causal relationships between NPWT modality and healing outcomes. Second, the sample size was modest, which may reduce statistical power and generalizability. Third, follow-up duration was short, preventing the assessment of long-term recurrence rates. Factors such as patient adherence, nutritional status, and presence of infection, all of which can impact healing, were not uniformly controlled. In addition, resource constraints limited access to continuous NPWT in all cases, reflecting real-world variability in wound care delivery. Finally, cost considerations, although not the primary outcome, remain an important barrier to widespread adoption of continuous NPWT in low-resource settings.

Table 6 summarizes key clinical and experimental studies that support the efficacy, biological rationale, and cost-effectiveness of NPWT in DFUs. The evidence consistently favors continuous NPWT for faster healing, reduced complications, and better patient outcomes, especially when combined with optimal offloading and glycemic control.

**Table 6:** Summary of key supporting studies for NPWT in diabetic foot ulcers NPWT: negative pressure wound therapy

Author(s) [Ref]	Key focus	Findings
Armstrong et al. [1]	NPWT vs. moist therapy (RCT)	Faster healing, fewer complications
Blume et al. [2]	NPWT in DFUs (RCT)	Higher closure rate
Apelqvist et al. [3]	Cost-effectiveness of NPWT	Reduced overall cost
Gentzkow et al. [4]	Dermagraft (human dermis) for DFUs	Promotes healing in chronic ulcers
Veves et al. [5]	Graftskin (human skin equivalent)	Effective in noninfected neuropathic DFUs
Steed [6]	rhPDGF for diabetic ulcers	Improved healing outcomes
Marston et al. [7]	Dermagraft efficacy	Improved healing in chronic DFUs
Lavery et al. [8]	Reducing dynamic foot pressure	Offloading helps reduce ulcer progression
Fleischli et al. [9]	Pressure reduction strategies	Reduced pressure enhances healing
Eginton et al. [10]	NPWT vs. gauze dressing	Faster healing, fewer infections
Banwell [11]	NPWT mechanism and wound care	Supports NPWT in clinical practice
Peters et al. [12]	Electrical stimulation in DFUs	Adjunctive benefit in healing
Wagner [13]	DFU severity classification	Aids in risk stratification and treatment
Saxena et al. [14]	Cell response to NPWT	Promotes proliferation and angiogenesis
Greene et al. [15]	NPWT and angiogenesis in chronic wounds	Increases angiogenesis, reduces MMP expression

## Conclusions

This study demonstrates that continuous NPWT yields better outcomes than intermittent NPWT in managing Wagner Grade 1 and 2 DFUs, with faster healing, lower infection rates, and fewer amputations. Predictors of successful healing, such as good glycemic control, absence of infection, and lower ulcer grade, underscore the need for a comprehensive, individualized approach. Given the growing burden of diabetes, continuous NPWT should be prioritized, especially in resource-limited settings where efficiency and cost-effectiveness are essential.
